# Induction of Experimental Peri-Implantitis with Strains Selected from the Human Oral Microbiome

**DOI:** 10.3390/biomedicines12040715

**Published:** 2024-03-22

**Authors:** Diana Larisa Ancuţa, Diana Mihaela Alexandru, Maria Crivineanu, Cristin Coman

**Affiliations:** 1 Faculty of Veterinary Medicine, University of Agronomic Sciences and Veterinary Medicine, 050097 Bucharest, Romania; diana.larisa.ancuta@gmail.com (D.L.A.); maria_crivineanu@yahoo.com (M.C.); comancristin@yahoo.com (C.C.); 2 Cantacuzino National Medical Military Institute for Research and Development, 050096 Bucharest, Romania; 3 Center of Excellence in Translational Medicine, Fundeni Clinical Institute, 022328 Bucharest, Romania

**Keywords:** peri-implantitis, rats, oral microbiome, *Aggregatibacter actinomycetemcomitans*, *Fusobacterium nucleatum*, *Streptococcus oralis*

## Abstract

Peri-implantitis (PI), the most widespread condition in the oral cavity, affects patients globally; thus, advanced research in both in vitro and in vivo studies is required. This study aimed to develop peri-implantitis in the rat model by oral contamination with bacteria responsible for PI in humans. The study was carried out in three stages: the extraction of the maxillary first molar to reproduce the human edentation, the mounting of the implant, and finally, the contamination of the device by gavage with *Aggregatibacter actinomycetemcomitans*, *Fusobacterium nucleatum* and *Streptococcus oralis*. The hematological examinations showed statistically significant increases for WBCs (white blood cells), Hb (hemoglobin), RBCs (red blood cells), MCH (mean corpuscular hemoglobin), MCHC (mean corpuscular hemoglobin concentration), and PLTs (platelets), but especially for the level of neutrophils and lymphocytes, and the systemic immunoinflammatory index completed the picture related to the inflammatory response triggered as a result of the activity of microorganisms pathogens on oral tissues. By examining the liver and kidney profile, we hypothesized that peri-implantitis is associated with systemic diseases, and the histopathological examination showed peri-implantitis lesions characterized by a marked inflammatory infiltrate with numerous neutrophils and lymphocytes. By corroborating all the results, we successfully developed a rat peri-implantitis model using a mixed bacterial infection through the oral gavage technique.

## 1. Introduction

Peri-implantitis (PI) is a pathological condition of the tissues surrounding the implants induced by the bacterial biofilm characterized by inflammation and progressive resorption of the bone support until the loss of contact with the implanted device.

Dental implants significantly improve the quality of life, but the location is prone to bacterial infections. A particular risk is the biofilm that develops on the implant’s surface, which acts on the surrounding tissues without appropriate treatment [1]. Bacterial species capable of forming the oral biofilm are classified into early colonizers (*Streptococcus* and *Actinomyces spp*.) and late colonizers, which include bacterial strains belonging to the “red complex,” as in periodontal disease (PD). The integration of bacteria into the biofilm largely depends on *Fusobacterium nucleatum* (F.n), which is one of the most frequently isolated bacteria from dental plaque. It is a “bridge” organism for colonizers through its ability to co-aggregate various microorganisms [2].

In many research studies, it has been hypothesized that the etiology and pathogenesis of PI are similar to PD; therefore, the therapeutic approach is similar. However, most of the time, the applied protocols cannot regenerate the affected tissues or achieve the reintegration of the implant in bone [3]. Clinically, PI is produced by several factors related to the patient, implant type, or placement site.

The complexity of the disease is difficult to understand from clinical data alone, and for this purpose, further studies involving animal models are needed. In recent years, several protocols have been tried to induce PI in rodents because they allow researchers to conduct studies with greater efficiency, resulting in reduced costs and shorter recovery periods [4].

As with PD, the histological features of PI refer to inflammation of the gingival tissue, peri-implant pocket formation, and bone resorption on the circumference of the implant. Clinically, these are assessed by increased depth on probing, bleeding or suppuration, edema/erythema, and decreased bone density seen on imaging examinations [5].

In vivo, PI research is classified into studies related to disease pathogenesis (tissue regeneration and systemic interaction) and studies focused on developing therapies. For the investigation of the mechanisms regarding the onset, progression, and healing of PI, rodents have the advantage of forming considerable samples compared to large animal models, which has also made it possible to investigate various systemic diseases (diabetes mellitus, xerostomia) as well as their interaction with PI. Findings in this direction have provided insight into the clinical management of PI, even though the small size of these animals limits the possible avenues for applying therapeutic protocols [6]. When new therapies for PI are tried, the animals are either before or in the entire evolutionary process of the disease [7], so we can comment on the effects of preventive treatments for regenerating peri-implantation tissues. Therefore, it is necessary to pursue the reparative potential of a therapeutic protocol after the diagnosis of PI has been established with certainty. *Porphyromonas gingivalis* is referred to as the ‘key pathogen’ for the development of IP and periodontitis [8]. It favors the survival of other bacteria such as A.a or F.n [9], and in experimental models, P.g, F.n and A.a have been used both alone [10,11] and in mixed infections [12,13] to induce PI.

For the in vivo evaluation of a new antibacterial treatment for PI, a disease-specific setup that allows reliable effects analysis is required. Therefore, this study aimed to develop an animal model of PI by the method of oral contamination with *Aggregatibacter actinomycetemcomitans* (A.a), *Streptococcus oralis* (S.o) and *Fusobacterium nucleatum* (F.n). Rats were used to facilitate ease of handling and upkeep as well as to accommodate a sufficiently ample population for inducing disease. 

## 2. Materials and Methods

### 2.1. Ethics Statement

This study adhered to ethical principles and complied with the regulations outlined in the E.U. Directive 63/2010 concerning the care, use, and protection of animals utilized for scientific purposes. Animal experiments were conducted at the Băneasa Animal Facility, within the Preclinical Testing Unit of the Cantacuzino National Medical-Military Institute for Research and Development, Bucharest (CI), which is duly authorized as a user by the appropriate regulatory body. Approval for the study was obtained from the Ethics Committee of the Faculty of Veterinary Medicine Bucharest under number 25/15.06.2022, as well as from the Directorate of Veterinary Health and Food Safety Bucharest, with project authorization number 27/29.08.2022. All measures were taken to minimize animal suffering for disease induction, clinical monitoring, and sampling (Supplementary Material) [14].

### 2.2. Study Design

This study aimed to develop peri-implantitis in the rat model by oral contamination with bacteria responsible for PI in humans, and it was carried out in three stages: the extraction of the maxillary first molar to reproduce the human edentation, the mounting of the implant, and finally, the contamination of the device by gavage with A.a, F.n and S.o, concentration 10^9^ CFU/mL, 5 days/week, 6 weeks. For this experiment, we used 20 male Wistar rats, weighing 350–400 g at the start of the study. Dental extraction required approaches and instrumentation similar to the human dental technique, and the period required to regenerate the dental alveolus was 30 days. This was followed by the implantation of the device on the edentulous site using specific equipment, and we also allowed 30 days for integration, which was a stage preceded by the exposure of the implant to the bacterial action and the follow-up of the clinical evolution [14].

### 2.3. Processing of Bacterial Strains Selected for Study

In this study, as in a previous study [14], we utilized A.a (ATCC 29522) serogroup b, provided by the CI bacterial strain bank and sourced from a mandibular abscess. Revitalization of the A.a strain involved inoculating 1 mL of the bacteria into a tube containing Schadler broth medium, which was followed by a 24 h incubation period at 37 °C under anaerobic conditions (80% nitrogen, 10% carbon dioxide, and 10% hydrogen). The density of both the unseeded medium tube and the 24 h suspension was quantified using a densitometer (Densitometer McFarland Biosan DEN-1, Riga, Lithuania), with the difference establishing the concentration of A.a, which was recorded at 10^9^ CFU/mL. Cryotubes containing the revitalized A.a culture were then stored at −80 °C and utilized to prepare daily inoculums for oral contamination, whereby one tube from the “mother” culture was used for each inoculation. Additionally, F.n (ATCC 25586), obtained from a cervicofacial lesion, and S.o (DSM 20627), isolated from the oral cavity of a human patient, were also acquired from the CI bacterial strain bank. The preparation of the inoculum for F.n and S.o followed identical procedures as outlined for A.a, including the use of the same culture media, cultivation conditions, and steps to establish inoculum density.

### 2.4. Animal Selection

Experimental implantations were performed in 20 male Wistar rats, which were obtained from the SPF (Specific Pathogen Free) Animalarium of CI. Throughout the duration of the study, rats were accommodated in conventional housing conditions with unrestricted availability of food (NCG diet manufactured by the CI Compound Feed Factory) and ad libitum access to water. The overall health condition of all animals underwent daily assessments. Specific to the implants, health status documentation occurred once every two days throughout the entirety of the experiment, commencing from the initial surgical procedure, and body weight was monitored every 14 days [14].

Human endpoint criteria were determined prior to the start of the study. They included the following conditions: weight loss of 20% or more at any point in the experiment that would have resulted in immediate animal euthanasia, loss of implants, or death before the final day. To minimize animal stress, the animals were housed in groups of five per cage and identified by a tag on which data related to species, line, age, sex, and cage number were specified, according to CI’s internal procedures.

### 2.5. The Type of Implant Used in the Experiment

**Figure 1:**
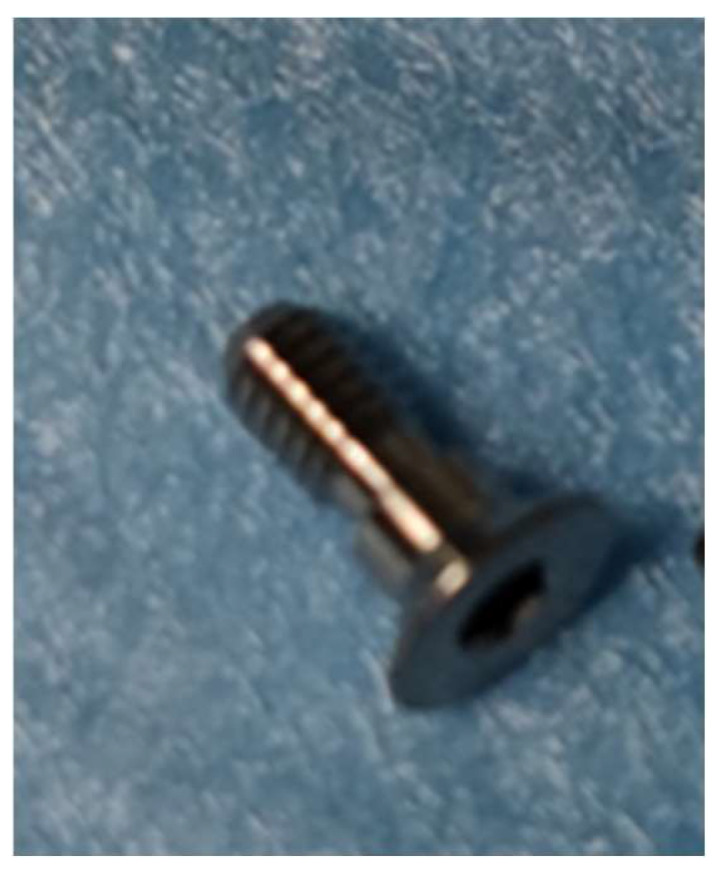
Titanium implants used in the study.

Twenty titanium implants were involved in the experiment, which were donated by the study director of another project that used dental implants (Zimmer Dental Implant, Winterhur, Switzerland). The devices were machined in cylindrical form, and self-tapping provided a smooth surface at the implant neck with a diameter of 1 mm and a length of 4 mm (Figure 1).

### 2.6. The Method of Inducing Peri-Implantitis

**Figure 2:**
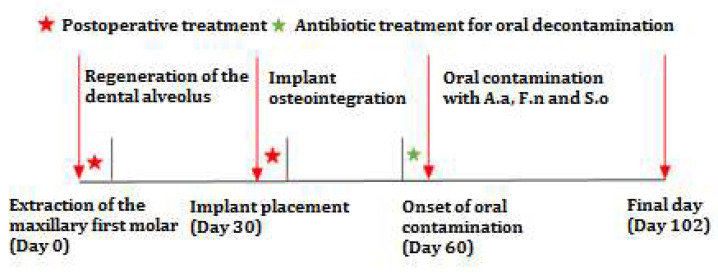
Timeline of the experimental peri-implantitis induction procedure.

The experimental procedure was carried out in three stages, as seen in Figure 2; the animals were generally anesthetized in the first two stages.

#### 2.6.1. Extraction of the Maxillary First Molar

Before surgery, animals were individually weighed to determine the anesthetic dose, and blood was collected for hematological and biochemical profiling at the beginning of the experiment. A control group was not used in this study, due to the reduction in animals used for scientific purposes, and the results of the analyses obtained after each applied procedure were compared with those at the beginning of the experiment.

**Figure 3:**
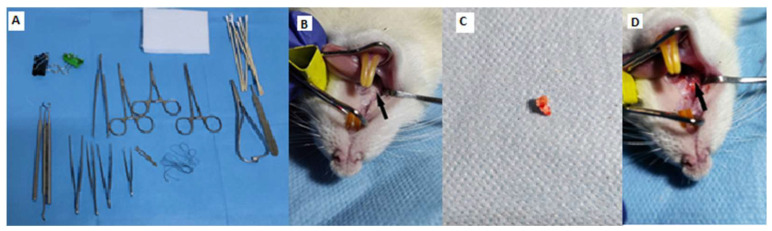
Stages of the dental extraction process. (**A**) instrument preparation; (**B**) positioning of the animal and identification of the molar: black arrow; (**C**) extracted tooth; (**D**) dental alveolus after extraction: black arrow).

Under general anesthesia, ketamine (0.5 mg/kg, Farmavet, Bucharest, Romania) and medetomidine (0.5 mg/kg, Biotur, Alexandria, Romania) administered intramuscularly, the rats were placed on the operating table in a dorsal–ventral decubitus position [14]. A buccal spacer was inserted between the upper and lower incisors. Utilizing a dental instrument designed for human application, the gum adjacent to the left first molar in the jaw was gently separated from the tooth. Through rotational movements in the axis of the tooth, its mobility was created until it was extracted (Figure 3). As the maneuvering space was relatively narrow, during the extraction procedure in some animals, only the tooth crown was detached; in this case, the retinal roots within the alveolus were extracted using surgical forceps to ensure a clear extraction site. Subsequently, the gingiva was sutured in a single point with a 4/0 nonabsorbable suture. 

Following the surgical procedure, the animals were administered an antidote to counteract the effects of anesthesia (atipamezole—0.02 mg/kg, Biotur, Alexandria, Romania), an antibiotic (enrofloxacin 10%, 2.5 mg/kg, Farmavet, Bucharest, Romania), and an anti-inflammatory agent (ketoprofen, 3 mg/kg, Dopharma, Ghiroda, Romania) for a duration of three days [14]. Subsequently, after a four-week period allowing for the healing of the extraction socket, radiological analyses were conducted (IVIS Lumina XRMS, Werner ROEDL—PerkinElmer, Traiskirchen, Austria) to assess the extent of bone regeneration.

#### 2.6.2. Mounting of Implants

After the four weeks required for tooth alveolus regeneration, the rats were anesthetized again using the same extraction protocol and positioned in the same recumbent position. Both the extraction procedure and the implant mounting were performed by the same surgeon.

**Figure 4:**
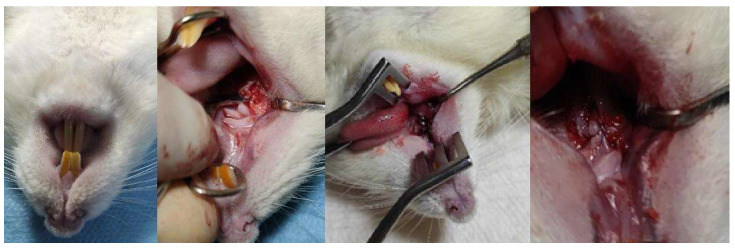
Dental implant mounting surgery in the rat.

The gum adjacent to the extracted molar was incised mesiodistally using a scalpel blade, after which it was detached from the underlying bone [15] (Figure 4). Once the submucosal bone was exposed, a 1 mm deep cavity was created using the drill attached to the X-Cube implant motor (Saeshin America, Irvine, CA, USA). The diameter of the bone defect was 1 mm, into which a titanium implant 4 mm long and 1 mm in diameter was screwed by hand screwing in a clockwise direction using a screw end from the implantology kit until it was no longer possible to move. The implant placement procedure involved meticulous positioning to prevent perforation of the buccal bone as well as the wall of the nasal cavity. Mobilization of the mucosa was achieved using a minimally invasive approach, incorporating a split flap technique around the implants to facilitate initial submerged healing. The incision was sutured with nonabsorbable suture material (PremiCron 6/0, B. Braun Surgical, SA, Rubi, Spain). Following implantation, a four-week period was allocated for healing and osseous integration.

#### 2.6.3. Oral Inoculation Procedure

The rats were pretreated daily with a combination of ampicillin (Antibiotice Iași, Iaşi, Romania) and kanamycin 25% (Farmavet, Bucharest, Romania), 20 mg/kg each, dissolved in drinking water for 5 days to eliminate the suppressed normal oral flora, which were protocol used in other studies in this field [12,16]. After decontamination, cotton swabs were used to collect samples to identify residual pathogens.

For each bacterial strain utilized for oral inoculation, the corresponding numbers of colony-forming units were previously determined from fresh 24 h cultures by densitometry. For oral inoculation, 10^9^ cells/strain/mL were used and administered in approximately 600 µL inoculum to each animal by oral gavage. Oral inoculation was performed in this manner 5 days per week for 6 consecutive weeks.

### 2.7. Methods for Evaluating the Induction of Peri-Implantitis

The evaluation of PI induction aimed at approaching the following methods:

Daily clinical examination (local and general): Monitoring of local and general clinical signs was carried out daily throughout the study by the same veterinarian, following, in particular, the appearance of the oral cavity. At this level, we ascertained the presence or absence of peri-implant bleeding or suppuration, the appearance of the gums, and the formation and depth of the pocket around the implant, which was measured with a periodontal probe (periodontal probe model CP 15, KOHLER Medizintechnik Gmbh, Stockach, Germany). The general condition of the animals was monitored by assessing body weight, appetite and posture.

Hematological examination (performed four times: after dental extraction, after mounting the implant, at the beginning of oral contamination, and the end of the experiment) was performed using the Idexx Procyte 5diff analyzer from venous blood collected from the retroorbital plexus, in vacutainers with EDTA (KIMA Vacutest, Arzergrande-PD, Italy). It is well known that oral diseases significantly influence systemic blood values. Therefore, the purpose of the analysis was to follow the polymorphonuclear cells (PMNs), white blood cells (WBCs), hemoglobin (Hb), red blood cells (RBCs), mean corpuscular hemoglobin (MCH), the mean corpuscular hemoglobin concentration (MCHC), the number of platelets (PLTs) as well as the systemic immunoinflammatory index (SII), which was calculated by the formula: SII = NEU × PLT/LYM where NEU means neutrophil count and LYM means lymphocyte count.

Biochemical examination (performed the exact same four times as the hematological examination) was performed from venous blood collected in tubes with lithium–heparin (KIMA Vacutest, Arzergrande-PD, Italy). Thus, with the help of the VetTest Idexx biochemistry analyzer, the general health profiles (GHPs) of the animals involved in the study were made. Since alkaline phosphatase (ALKP) is a protein associated with bone resorption, ALT (alanin-amino-transferase), AST (aspartate-amino-trasferase), and GGT (gamma-glutamyl-transferase) are enzymes whose values are evaluated to determine the liver damage developed in patients with periodontitis. The level of urea in the blood guides the level of tissue damage caused by inflammation periodontal disease, so we considered it appropriate to identify the involvement of parameters such as glucose, ALKP, ALT, cholesterol, urea (BUN), and creatinine(CREA) found in the GHP profile in the induction of PI.

Microbiological examination was performed after treatment with ampicillin–kanamycin and at the end of the study. The rats were given ampicillin and kanamycin at the beginning of the experiment for 5 days to obtain the decontamination of the oral cavity, and samples were taken and analyzed in MaldiTof for identification to check the types of residual bacterial strains. Also, at the end of the oral contamination period, we checked the presence of the microorganisms under study using the same method.

The MaldiTof technique for identifying bacterial strains allows the rapid and high-accuracy identification of anaerobic bacteria, being recently preferred instead of conventional methods that are more difficult to perform and consume time and material resources. MaldiTof involves using mass spectrometry, which helps to understand the biological system by analyzing the bacteria’s protein, amino acid, carbohydrate, or lipid molecules. Thus, after the samples were collected from the rats at the beginning and end of the experiment, they were seeded on a Schadler culture medium (liquid and solid). From the fresh 24 h colonies, the oral flora resistant to antibiotic therapy and the one developed during the experiment were identified. The final objective of the analysis was to track the presence of A.a, F.n, and S.o alongside the commensal microorganisms.

Radiological examination was performed post-extraction, one month after implant placement, and at the end of the study. Establishing the diagnosis of PI, the stage and the evolutionary phase are ensured by the corroboration of clinically expressed signs and imaging techniques (intraoral or panoramic radiographs) to determine the degree of bone loss. Even though micro-computed tomography is a modern analysis method, classic radiography is still a valid and reliable tool for detecting peri-implant disease. At the beginning and end of the experiment, in order to appreciate the impact of the bacterial action on the bone support, we evaluated the animals radiographically by exposure to the In Vivo Imaging IVIS Lumina XRMS device (Werner ROEDL—PerkinElmer, Traiskirchen, Austria), which allows obtaining sensitive multispectral images in just 10 s. For such an analysis, it is necessary to anesthetize the animals so that the images and their quality are superior.

The rats were humanely euthanized after 102 days of the experiment by anesthetic overdose, according to the regulations in force. Animals were euthanized within seconds, which was confirmed by cardiac arrest and the cessation of involuntary reflexes. The implanted jaws were harvested and conditioned in 10% formaldehyde containers for histopathological analysis.

Histopathological analysis: The collected samples were fixed in 10% formaldehyde and then decalcified in a decalcifying solution (Histo-Decal, Pantigliate, Italy) for 14 days. After the decalcification period, the samples were processed for paraffin embedding by making serial sections of 5 µm, which was followed by hematoxylin–eosin staining to be analyzed under an optical microscope (DM 4000B, Leica, Wetzlar, Germany). Each sample was blindly examined by a specialist histologist who followed the influx of inflammatory infiltrate and the integrity of the alveolar bone support. Samples from healthy animals were also examined to identify the regular appearance of rat oral alveolar tissues and bone.

Statistical analysis was performed using Prism 9 for Windows software (GraphPad LLC, Boston, MA, USA). The sample size was calculated a priori using the Pearson correlation coefficient according to G*Power software (version 3.1.9.7; Heinrich-Heine-Universität Düsseldorf, Düsseldorf, Germany) [17]. To compare the data, the one-way ANOVA function was used, and a value of *p* < 0.05 was considered statistically significant. Regarding the analysis of the data obtained from the hematological and biochemical examination, we compared the results obtained after the extraction of the molars, after the installation of the implants in the edentulous spaces, before oral contamination, and at the end of the period of oral contamination, using one-way ANOVA function, multiple comparisons, the Bonferroni test.

## 3. Results

### 3.1. Results Obtained from Clinical Evaluation

**Figure 5:**
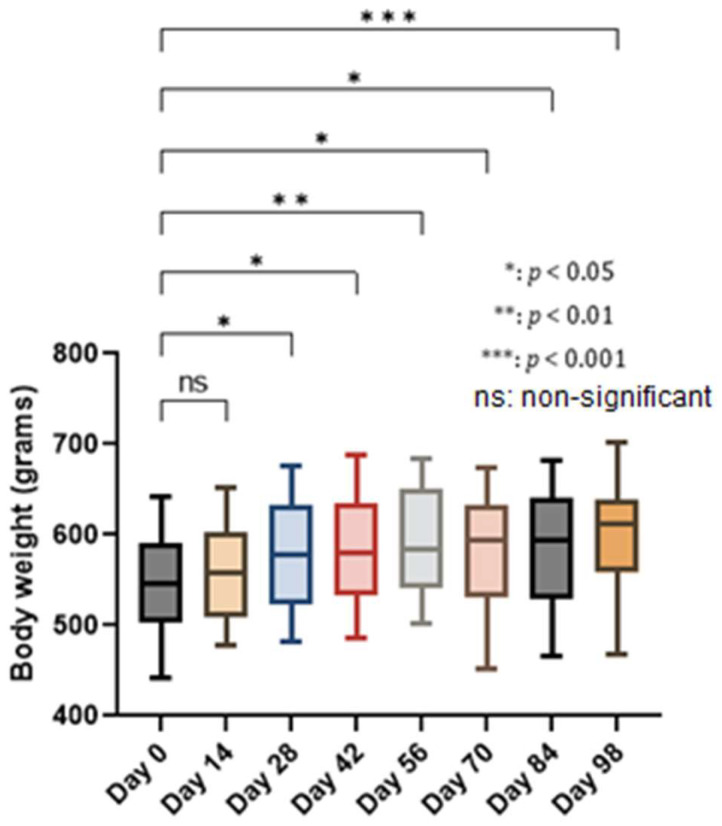
Evolution of body weight in the process of peri-implantitis development: an increase in body weight after extraction can be observed, starting from day 14, maintained in an upward trend until almost the day of implant installation (day 56), when *p* < 0.01. After the installation of the implant, a slight decrease in weight was recorded, which was recovered until the end of the study. Comparing the weight of the animals at the beginning with that obtained at the end of the experiment, it can be said that it was slightly influenced, especially after the surgical interventions, but *p* < 0.001 between day 0 and day 98 suggests the favorable evolution of animal welfare during the study.

Surgical procedures were completed without incident both after extraction and after implant placement. All rats started to eat normally so that the body weight did not register negative values but on the contrary. The increase in the weight of the animals had statistically significant values from the second week of the experiment and remained so until the end with a value of *p* < 0.001 (Figure 5).

Macroscopic examination was difficult due to the reluctance of the animals, but after both interventions on the oral tissues, the gingiva healed within about two weeks. Detailed notes on the health status of the peri-implant mucosa were taken after the completion of oral decontamination based on gingival indices [18] where 0 = normal mucosa, 1 = mild inflammation (slight color changes, slight edema, no bleeding) 2 = moderate inflammation (redness, edema, bleeding on probing), 3 = severe inflammation (marked redness and edema, ulceration, spontaneous bleeding tendency).

**Figure 6:**
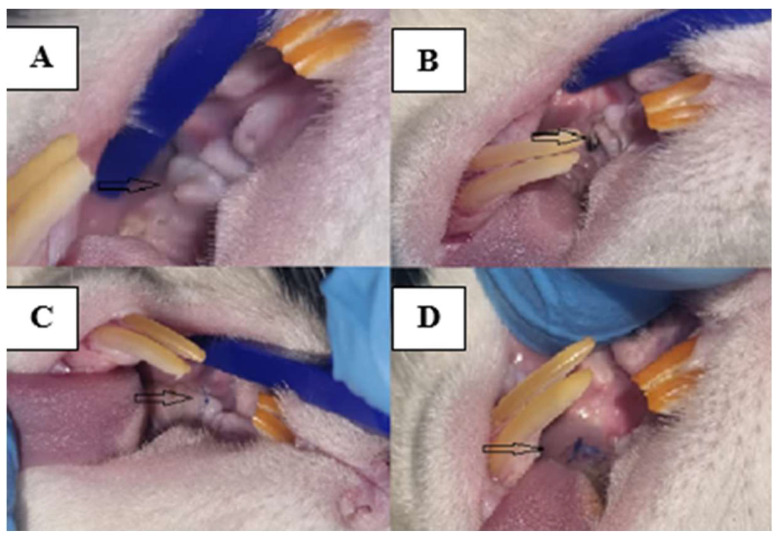
Clinical aspect of the implanted zone at the beginning of oral contamination (indicated by the arrow with black outline). The vast majority of animals (**A**,**C**,**D**) have healthy gums, where the suture line has been preserved. In only one case (**B**), the implant appeared exposed, with the gingival margin, with a normal appearance, molded on the implant.

**Figure 7:**
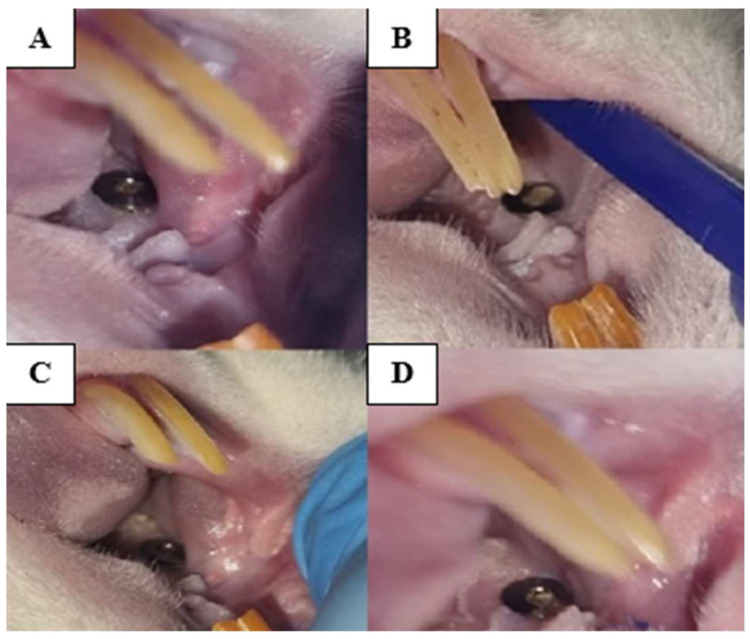
Clinical aspect of the implant zone of 3 weeks of oral contamination and observation of implants. The implants appear uncovered (**A**–**D**), covered with plaque and food debris (**A**–**D**), and the gingival margin has a changed color (**C**).

**Figure 8:**
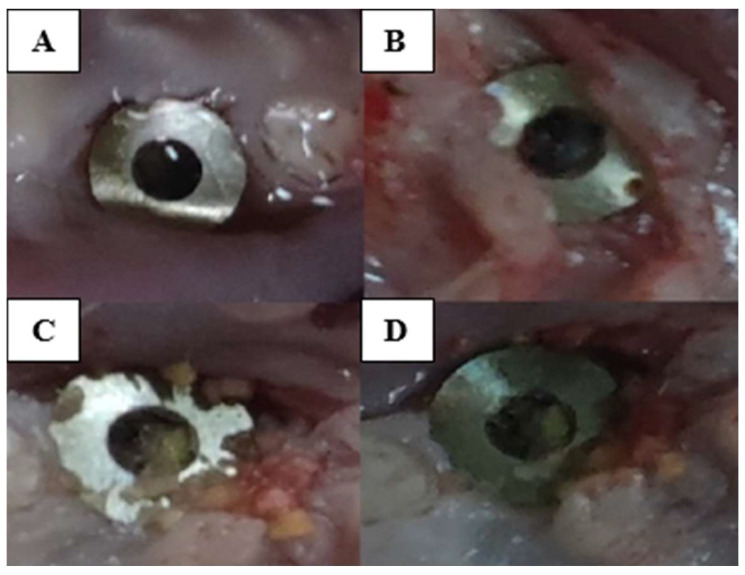
Clinical aspect of the implanted area at the end of the experiment. Uncovered implants, covered with plaque and food debris (**A**–**D**), frayed gingival margin (**A**,**B**,**D**), bleeding around the implants (**A**,**C**,**D**).

Therefore, over 50% of the animals in which the implant was retained until the end of the study fit into a score of 2. Although the gingiva was sutured over the implant, minor breaches were created with the onset of contamination so that at the end of the study, fully exposed implants, with retracted gingiva, pinkish-gray in color, bacterial plaque accumulated all around and reduced bleeding on probing could be observed (Figure 6, Figure 7 and Figure 8).

### 3.2. Results Obtained from Hematological Examination

**Figure 9:**
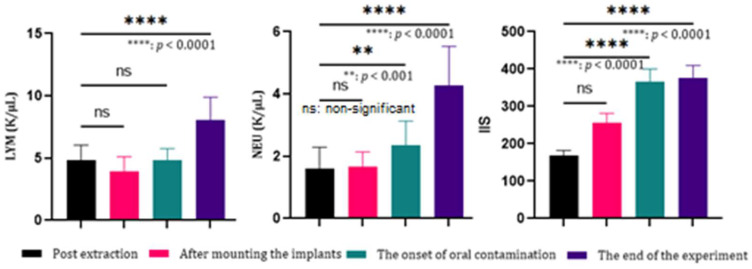
Evolution of neutrophils, lymphocytes, and the systemic immunoinflammatory index in the development of peri-implantitis in rats.

The relationship between PI and changes in hematological parameters has yet to be investigated in experimental studies. Therefore, we evaluated the dynamics of the complete blood count after extraction of the maxillary first molar, after implant placement, at the onset of oral infection, and at the end of the study. Interest was focused on the analysis of the parameters of the white line where lymphocytes, neutrophils, and the systemic immunoinflammatory index had *p* < 0.0001, a value with strong statistical relevance, especially after the onset of oral contamination (Figure 9).

The observed hematological changes were associated with PI as there were substantial differences among WBC, Hb, RBC, MCH, MCHC, and PLT after the onset of oral contamination. The body’s response to bacterial action consisted of a marked increase in WBC, MCH, and PLT and a decrease in Hb and RBC values, the analysis confirming that PI is closely related to an increased number of leukocytes. These results are also consistent with the evidence obtained in other PD induction studies [14], which reflect the inflammatory response of the animal organism to PI (Table 1).

### 3.3. The Results of the Biochemical Examination

We hypothesized that, like in periodontal disease, bacteremia combined with immune system stimulation plays a role in the development of systemic disease. In our research, however, we did not find any evidence that PI would be directly related to pathological changes developed in other distant organs. Therefore, the biochemical examination aimed to analyze the consequences of experimental PI on biochemical indicators that reflect organ health.

**Figure 10:**
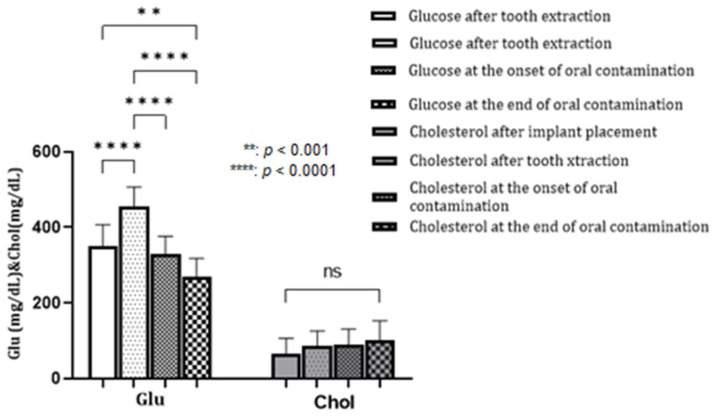
Dynamics of blood glucose and cholesterol levels.

Biochemical values were divided into three variables: kidney function, liver function, and metabolic function (blood glucose and cholesterol level). The kidney subset included values for BUN and CREA concentration, the liver function subset analyzed ALP, ALT, and the last subset was for blood glucose and cholesterol levels. Regarding the glucose values, we observed a significant increase in the concentration with statistically significant relevance after the implant installation (*p* < 0.0001) maintained until the middle of the period of oral contamination, after which a decrease followed, so that on the final day, *p* < 0.001, compared to day 0, as seen in Figure 10. Cholesterol values, although showing increasing levels throughout the experiment, did not reach statistical significance, so we do not consider PI to have any influence on this parameter.

**Figure 11:**
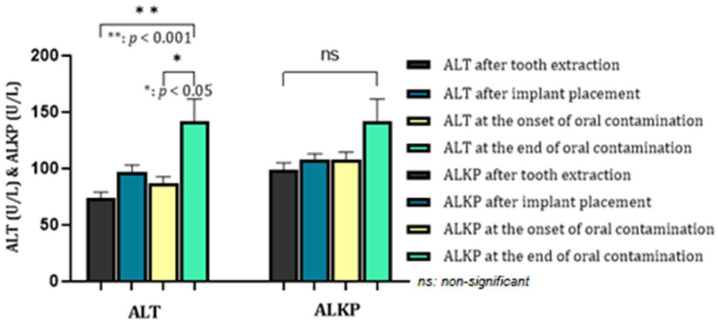
Dynamics of ALT and ALKP in the process of peri-implantitis development.

If in periodontal disease, ALKP shows changes in bone metabolism, in PI, it did not show significant oscillations. However, especially at the end of the experiment, the enzyme values were noticeably higher (Figure 11). ALT, at the end of the study, had a higher level (*p* < 0.001) than the experiment’s beginning.

**Figure 12:**
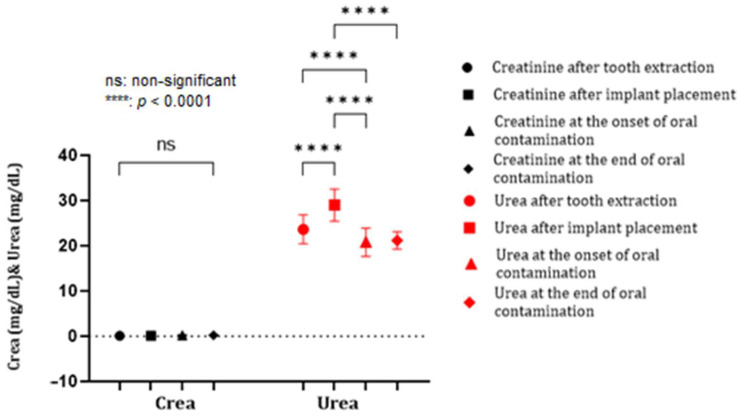
The dynamics of creatinine and urea in the blood.

The renal profile expressed upward changes related to the level of blood urea with a solid statistical relevance, observed after the installation of the implant (*p* < 0.0001), followed by essential decreases throughout contamination of the oral cavity (Figure 12).

### 3.4. The Results of the Microbiological Examination

At the beginning of the study, an oral flora rich in species of *Staphylococcus sciuri, Staphylococcus xylosus,* and *Micrococcus luteus* was identified, which are flora that showed resistance to the antibiotics administered in the drinking water. The samples from saliva and sac around the implant, collected at the end of the experiment, revealed several bacterial species, among which we also found the strains of A.a, F.n, and S.o. This indicates the stability of bacteria on the implanted material. However, due to the abundance of microorganisms, a quantitative analysis of the species selected for our study could not be performed.

### 3.5. The Results of the Radiological Examination

To check the condition of the alveolar bone, we performed conventional radiographs 30 days after the extraction of the maxillary molar, 30 days after implant placement, and at the end of the experiment. The objectives of the three exposures were to evaluate the regeneration of the alveolus after extraction, to follow the integration of the implant, and finally to verify the stability of the device after contact with the bacteria responsible for PI.

**Figure 13:**
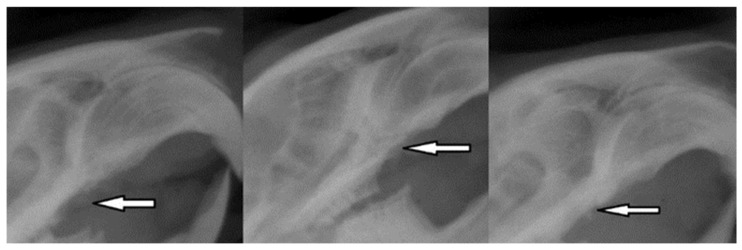
Radiographic images 30 days after tooth extraction showing alveolar bone regeneration (white arrow).

After the first exposure, the radiological results showed optimal bone regeneration, the uniform implant bed, and bone density within physiological limits (Figure 13).

**Figure 14:**
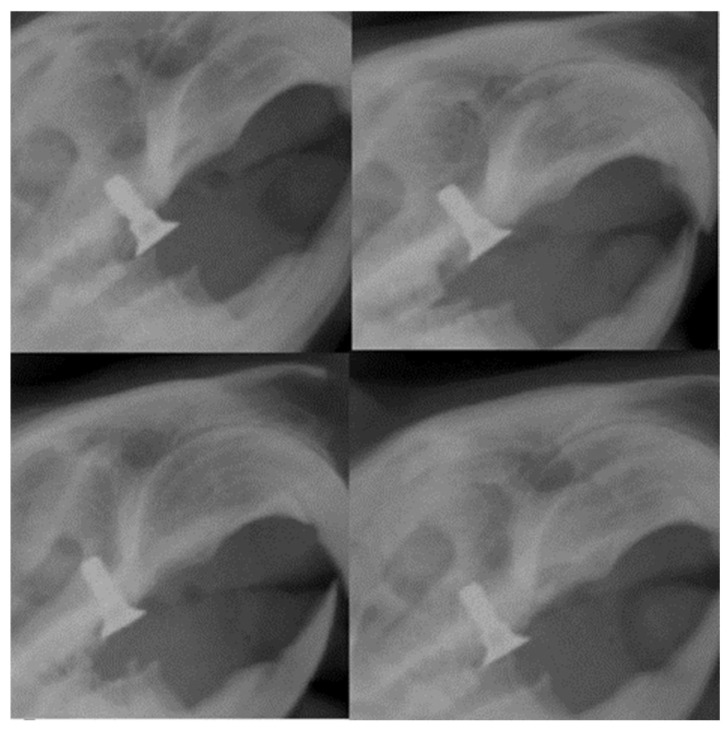
Radiographic images 30 days after placement of the implants in rats showing their parallel position with the rest of the teeth and the height concerning the dental plaque.

Radiological assessment is critical after implant placement, as the alveolar ridge’s height, width, and contour must be determined precisely. In the case of our study, the results showed an excellent fixation at the level of the alveolar ridge, the implant being in a parallel position with the remaining molars on the dental arch (Figure 14).

**Figure 15:**
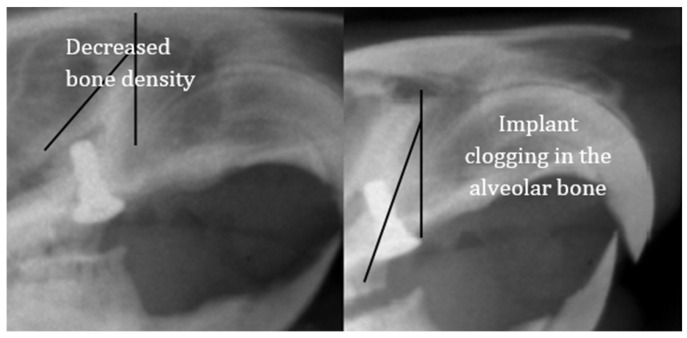
Radiological appearance of the implants at the end of the period of oral contamination.

In peri-implant disease, radiographic images can assess the height of the alveolar bone in relation to the implant structure. In the case of our experiment, after the completion of the six weeks of bacterial contamination, it was possible to observe the displacement of the implants from the position parallel to the roots of the collateral teeth and a reduction in the bone density at the edge of the alveolar ridge (Figure 15).

### 3.6. The Results of the Histopathological Examination

**Figure 16:**
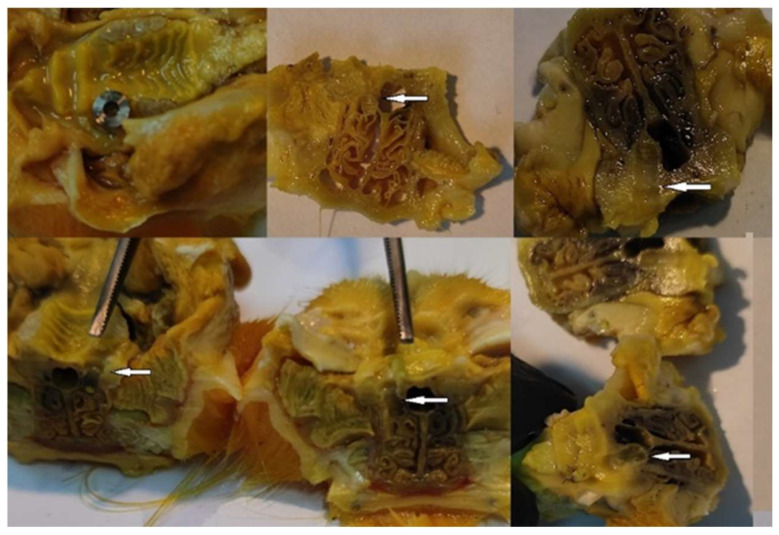
Sections through the harvested piece after decalcification—the white arrow indicates where the implant was inserted.

**Figure 17:**
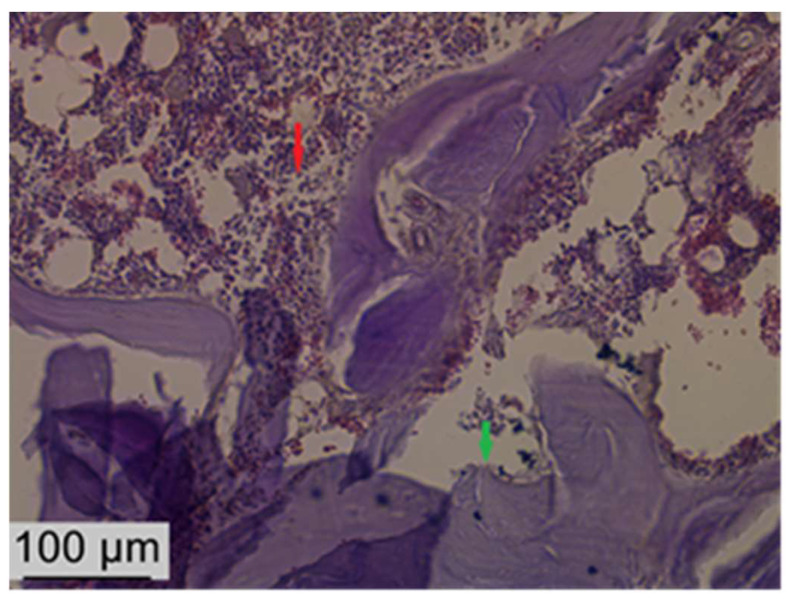
Histological features at the alveolar bone level: inflammatory infiltrate rich in lymphocytes and neutrophils, osteoblasts (red arrow), and disorganized bone matrix (green arrow). Hematoxylin–eosin staining, ob 20×.

**Figure 18:**
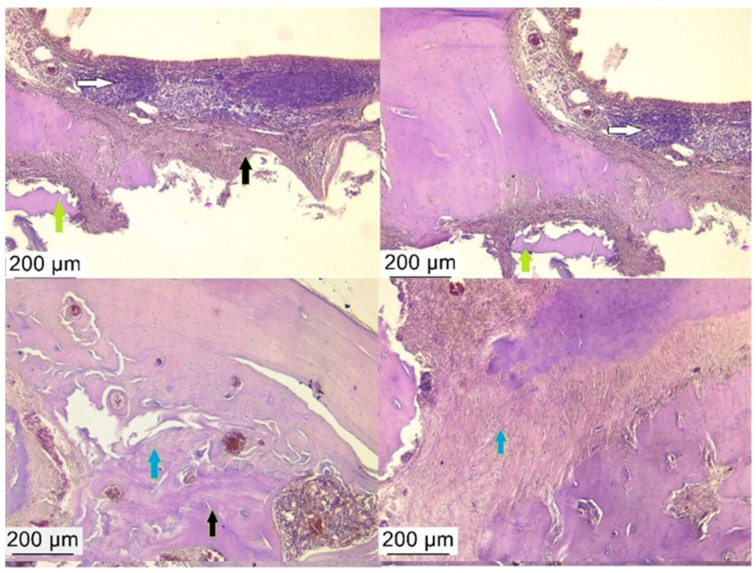
Pronounced inflammatory reaction at the gingival margin (white arrow) accompanied by reorganization of connective tissue and bone support (black arrow) alongside a rich infiltrate of collagen fibers and fibrous tissue (blue arrow). The green arrow shows the detachment of bone fragments, which is delimited by a marked inflammatory reaction. Hematoxylin–eosin staining, ob. 10×.

After six weeks of bacterial contamination, massive neutrophil and macrophage infiltrations were present in the oral connective tissue of rats. The specimens showed numerous fibroblasts and neutrophils in the peri-implant groove, which were characterized by extensive epithelial layer ulceration. The number of collagen fibers was also increased, as was the number of osteoclasts, which indicates an intense activity in the bone resorption process. The sulcular epithelium appeared strongly keratinized, and numerous fibroblasts invaded the peri-implant tissues. The host’s response was characterized by the appearance of an inflammatory infiltrate consisting of numerous lymphocytes, plasma cells, and neutrophils (Figure 16, Figure 17 and Figure 18).

## 4. Discussion

Both invasive and non-invasive methods can assess the stability of an implant. Invasive techniques refer to the pull/push test [19,20,21], the disassembly test [22], or histological analysis [23,24]. These methods are not suitable for clinical practice; therefore, the refinement of non-invasive methods is required [25,26], namely, the imaging approach after implant insertion [27], resonance frequency analysis [28], or thorough clinical examination. For preclinical studies, the corroboration of non-invasive and invasive methods could provide favorable results applicable in the clinical area. 

Animal models are the ideal solution for understanding the pathophysiological processes of peri-implant disease or testing innovative devices because they offer the possibility of following each pathogenic stage and verifying osseointegration in a living organism [29]. The medical world is continuously searching for the best animal model and testing protocol to increase the reliability of experiments [30] so that they are reproducible [31]. The ISO/TS_22911:2016 guideline offers recommendations for the preclinical assessment of implants, encompassing morphological, radiographic, and histopathological perspectives [32].

Osseointegration refers to the acceptance of the implant by the living bone support [33]. The implant–bone interface is an area of significant interest that depends on the type of implant, the nature of the alveolar bone, the surgical approach applied, and the post-interventional measures applied. Any of these factors can have a negative impact on the body’s acceptance of the device. Therefore, it is vital to know the elements that could influence the success of osseointegration. The most common cause of implant loss is oral hygiene, which favors the development of bacterial biofilms. They are made up of a great diversity of strains. However, research has classified microorganisms into red, orange, or yellow complexes, depending on their aggressiveness or colonization capacity. In summary, the main bacterial species found in the oral cavity of patients with peri-implant disease are represented by *Porphyromonas gingivalis* (P.g), *Aggregatibacter actinomycetemcomitans* (A.a), *Tannerella forsythia* (T.f), and *Treponema denticola* (T.d) to which is added the main binder called *Fusobacterium nucleatum* (F.n). 

Through this study, we intended to create an animal model for testing dental implants that approximates the bone microstructure of the human jaw. Moreover, in this model, we induced the disease state by contamination with bacterial strains responsible for PI in humans.

Through the extraction of the maxillary left molar, our objective was to simulate the edentulous region characteristic of a human patient requiring a dental implant. Several researchers searched for the right place in the oral cavity to obtain maximum implant stability; thus, the alveolar ridge of the diastema region was reported [34] or, as in our case, the extraction site of the maxillary first molar [35]. Through the surgical approach applied in our study, covering the implant with gingival tissue, we limited the action of bacteria at the level of the implant. The success of the integration of the implant in the bone still depends on its exposure in the oral cavity, and in the case of rats, where possible actions on the implants are uncontrollable, the applied technique, with a covered implant, proved to be useful. Later, after the microorganisms began to act on the oral tissues, the revelation of the implants strengthened the hypothesis about the action in the depth of the tissues of the strains of A.a, F.n and S.o. Given the prevalence of dental implantation procedures among the elderly population, the age of the rats was deliberately selected to align with an elderly status, which was typically considered after 20 weeks of age. Aging exerts a multifaceted impact on various cellular processes, including immune responses, thereby potentially influencing the healing outcome of bone injuries, whether spontaneous or induced [36]. Young and healthy animals are used in research, which only provides the best results if the information is to be translated into clinical trials. It is well known that advanced age and comorbidities influence vascular function, causing limited angiogenic responses in human patients [37], so it is necessary to induce PI, considering these particularities related to the physiological condition of the living organism.

The pattern of PI achieved in the experiment depended on the host’s inflammatory response in the soft tissues and bone loss around the implant. Polymicrobial infection with A.a, F.n and S.o probably induced these pathological changes over six weeks. F.n frequently appears in the biofilm developed on dental implants. However, S.o, while associated with the healthy state of the periodontal tissues [33], has proven its synergistic action with both A.a and F.n. in the development of PI. Therefore, the animal model reproduced elements encountered in human PI and may be a valuable tool for future research of relevant pathological pathways of peri-implant diseases and new therapeutic approaches. 

PI is a condition characterized by a sequence of bone changes around the implant, bleeding on probing, and the development of peri-implant pockets [38]. In our study, signs of mucosal inflammation and bone resorption were observed along the way, providing clues to the establishment of PI by mixed oral bacterial contamination. Although the normal flora of the rats was temporarily suppressed by the antibiotic treatment given before the contamination period, it was not eliminated; it gradually recovered and contributed to the exacerbation of PI. Moreover, the implants themselves can change the microenvironment, so they are more prone to the attachment of external bacteria. The properties of the implant (e.g., roughness) influence the accumulation of microorganisms and the formation of bacterial plaque [39], resulting in the loss of bone support. Our concern was focused on the actual action of the bacteria and not on the type of implant, the chosen model being the one with the least influence on the progression of the disease.

The rat model in our research has many histopathological similarities with the human peri-implant lesion dominated by leukocytes and inflammatory infiltrate occupying more than half of the connective tissue portion [40]. In the present study, cellular infiltrates were present in connective tissues with variable distribution and not as pronounced as in humans, where massive bone loss is observed. Similar to the human situation or those reported in other studies [7,12], the most severe reaction in rats was in the apical area, where the tissue appeared infiltrated with inflammatory cells such as lymphocytes and neutrophils. The epithelium around the implant showed pathological signs of proliferation, and the bone mainly showed phenomena of bone lysis compensated by fibrous tissue. 

In 1966, the first attempt was made to induce periodontal disease in rats, and the results obtained showed massive inflammatory infiltrates in animals whose oral cavity was contaminated with bacteria compared to control groups where no bacterial strains were involved [41]. Since this study, oral lavage has been the primary method of inducing PD using bacteria such as A.a, P.g [42] or different combinations of microorganisms. To our knowledge, the present study is the first to apply A.a, F.n and S.o by oral gavage as colonizers to induce PI in rats. S.o. is a primary colonizer of dental plaque that expresses adhesins and forms a biofilm to which other microorganisms can adhere. F.n is also a common plaque bacterium and a bridge between the multiple species of the oral microbiome. The late colonizer, A.a, is a pathogen associated with both periodontitis and PI. Its presence in the biofilm structure provides a specific virulence on tissue destruction. In rats, A.a contributes to the development of inflammation of the gingival tissue, and together with the other bacteria, the bone loss at the level of the implant is greatly intensified.

In the present research, we selected three bacterial strains with a role in the development of PI in humans, which, administered by gavage to rats, adhere to the implant’s surface and induce an inflammatory response from the host. The microbiological examination at the end of the experiment highlighted the persistence of A.a, F.n, and S.o in the saliva samples of the rats. However, it was not possible to quantitatively determine the strains. A possible explanation for this limitation may be the diversity of the oral microbiome that may affect the body’s response to artificially introduced exogenous bacteria [43,44], thus resulting in high variability in detecting the strains involved in our research.

Analysis of the blood count allowed following the systemic effect of various challenges, such as implant placement surgery or experimental infection. The SII is a novel marker of inflammation and a predictive tool for the prognosis of tumors and immune response [45]. Clear associations were observed between SII and inflammatory conditions [46], which also correlated with the loss of bone density [47], and in our case, we observed an SII with upward evolution directly proportional to the period of oral contamination and the body’s response to consecutive inflammatory processes. The most important finding is that intraoral intervention can influence systemic blood values. Statistically significant increases were observed for WBC, Hb, RBC, MCH, MCHC and PLT but especially for the level of neutrophils and lymphocytes. Such results were also reported in other studies [48], encouraging careful analysis of blood count values in establishing the diagnosis of PI.

Peri-implant disease can also have effects on the systemic condition. Regarding PI, few studies have analyzed the correlation between it and conditions triggered in other organs. In our study, we aimed to evaluate the relationship by performing a biochemical examination and carefully analyzing the liver and kidney profile and glycemic values. This experiment’s increase in ALT, ALKP, and urea activity may be associated with PI as statistically significant values were recorded, especially at the end of the oral contamination period. Therefore, the hypothesis that PI causes inflammation in the liver and kidney parenchyma is worth exploring. The relationship between blood glucose values and PD has been established in humans. However, for PI, this association needed to be validated, as studies in other animal models also did not report conclusive results [49].

Animal studies facilitate knowledge of pathogenesis and enable a therapeutic exploration of human diseases. In the presented research, like Blank et al. [16], we can say that the 6-week period made possible the precise and reproducible induction of PI manifestations and implicitly supported possible therapeutic approaches in this critical phase of the disease. The use of animals in such studies requires ethical justifications. Therefore, the number of animals involved in the experiment was minimal for statistical differences. A trained staff performed the surgical protocols addressed, and the welfare of the animals was monitored daily. The introduction of human pathogenic strains into the mouths of rats resulted in an obvious clinical picture so that therapeutic strategies would have an effect in future smaller groups with the possibility of minimizing animal experiments.

This study has some limitations. First, the non-clinical study involved histological analysis commonly used in bone regeneration studies but lacked an analysis of the expression and distribution of bone regeneration proteins, such as osteocalcin and alkaline phosphatase, by immunohistochemical determinations or ELISA techniques. Secondly, analysis of parameters such as neutrophils or even alkaline phosphatase at the site of infection would have provided a better picture of their reactivity and direct connection with PI. Also, for the fact that we could not perform a quantitative analysis of the bacteria introduced in the study, we consider that all these limitations need further supplementation by future studies.

## 5. Conclusions

Mixed oral contamination with A.a, F.n, S.o has not been reported in any other specialty study, and the 6-week period was sufficient to observe the specific signs of PI. Tissue healing, bone regeneration, and the osseointegration of the implants occurred under normal conditions without affecting the general condition of the animals. As the action of the bacteria intensified, specific signs of inflammation appeared at the implanted site, which was an aspect also revealed by blood and imaging tests and histopathological examination. The inflammatory response and bone resorption are close to that observed in humans. Through the obtained results, we have successfully developed a rat model of PI using a mixed bacterial infection through the oral gavage technique. This can serve as a support for testing new therapeutic strategies to cure PI.

## Figures and Tables

**Table 1:** Evolution of blood parameters during the development of peri-implantitis in the rat.

Hematological Parameter (Mean Value ± SD)	Post-Tooth Extraction	After Implant Placement	Onset of Oral Contamination	End of Oral Contamination Period
WBC K/µL	6.86 ± 1.74	5.96 ± 1.40	6.96 ± 2.06	12.31 ± 3.077
HGB g/dL	16.71 ± 0.97	9.97 ± 2.11	15.73 ± 2.94	16.51 ± 0.95
RBC M/µL	9.45 ± 0.64	9.34 ± 1.26	8.47 ± 1.64	9.19 ± 0.61
MCH pg	17.70 ± 0.59	10.64 ± 1.81	18.60 ± 1.04	17.99 ± 0.79
MCHC g/dL	36.64 ± 0.74	20.67 ± 3.36	34.35 ± 1.33	35.76 ± 0.60
PLT K/µL	500.20 ± 171.02	607.28 ± 183.27	720.57 ± 193.57	681.26 ± 147.48

## Data Availability

Data are contained within the article.

## Supplementary Materials

The following supporting information can be downloaded at: https://www.mdpi.com/article/10.3390/biomedicines12040715/s1, Supplementary File S1: The ARRIVE guidelines 2.0: Author checklist.

## References

[1] Elter C., Heuer W., Demling A., Hannig M., Heidenblut T., Bach F.W., Stiesch-Scholz M. (2008). Supra- and subgingival biofilm formation on implant abutments with different surface characteristics. Int. J. Oral Maxillofac. Implant..

[2] Kolenbrander P.E., Palmer R.J., Periasamy S., Jakubovics N.S. (2010). Oral multispecies biofilm development and the crucial role of cell-cell distance. Nat. Rev. Microbiol..

[3] Roccuzzo M., Layton D.M., Roccuzzo A., Heitz-Mayfield L.J. (2018). Clinical outcomes of peri-implantitis treatment and supportive care: A systematic review. Clin. Oral Implants Res..

[4] Graves D.T., Fine D., Teng Y.T., Van Dyke T.E., Hajishengallis G. (2008). The use of rodent models to investigate host-bacteria interactions related to periodontal diseases. J. Clin. Periodontol..

[5] Renvert S., Persson G.R., Pirih F.Q., Camargo P.M. (2018). Peri-implant health, peri-implant mucositis, and peri-implantitis: Case definitions and diagnostic considerations. J. Periodontol..

[6] Ozawa R., Saita M., Sakaue S., Okada R., Sato T., Kawamata R., Sakurai T., Hamada N., Kimoto K., Nagasaki Y. (2020). Redox injectable gel protects osteoblastic function against oxidative stress and suppresses alveolar bone loss in a rat peri-implantitis model. Acta Biomater..

[7] Chew R.J.J., Lu J.X., Sim Y.F., Yeo A.B.K. (2022). Rodent peri-implantitis models: A systematic review and meta-analysis of morphological changes. J. Periodontal Implant Sci..

[8] Hajishengallis G., Lamont R.J. (2012). Beyond the red complex and into more complexity: The polymicrobial synergy and dysbiosis (PSD) model of periodontal disease etiology. Mol. Oral Microbiol..

[9] Heyman O., Horev Y., Mizraji G., Haviv Y., Shapira L., Wilensky A. (2022). Excessive inflammatory response to infection in experimental peri-implantitis: Resolution by Resolvin D2. J. Clin. Periodontol..

[10] Tzach-Nahman R., Mizraji G., Shapira L., Nussbaum G., Wilensky A. (2017). Oral infection with Porphyromonas gingivalis induces peri-implantitis in a murine model: Evaluation of bone loss and the local inflammatory response. J. Clin. Periodontol..

[11] Monasterio G., Castillo F., Astorga J., Hoare A., Terraza-Aguirre C., Cafferata E.A., Villablanca E.J., Vernal R. (2020). O-Polysaccharide Plays a Major Role on the Virulence and Immunostimulatory Potential of *Aggregatibacter actinomycetemcomitans* during Periodontal Infection. Front. Immunol..

[12] Sun J., Eberhard J., Glage S., Held N., Voigt H., Schwabe K., Winkel A., Stiesch M. (2020). Development of a peri-implantitis model in the rat. Clin. Oral Implants Res..

[13] Varon-Shahar E., Shusterman A., Piattelli A., Iezzi G., Weiss E.I., Houri-Haddad Y. (2019). Peri-implant alveolar bone resorption in an innovative peri-implantitis murine model: Effect of implant surface and onset of infection. Clin. Implant. Dent. Relat. Res..

[14] Ancuta D.L., Alexandru D.M., Crivineanu M., Coman C. (2023). Induction of Periodontitis Using Bacterial Strains Isolated from the Human Oral Microbiome in an Experimental Rat Model. Biomedicines.

[15] Ancuta D.L., Crivineanu M., Coman C. (2003). The development of a preclinical model for osteointegration of dental implants—A pilot study. Sci. Work. Ser. C Vet. Med..

[16] Blank E., Grischke J., Winkel A., Eberhard J., Kommerein N., Doll K., Yang I., Stiesch M. (2021). Evaluation of biofilm colonization on multi-part dental implants in a rat model. BMC Oral Health.

[17] Kang H. (2021). Sample size determination and power analysis using the G*Power software. J. Educ. Eval. Health Prof..

[18] Löe H., Theilade E., Jensen S.B. (1965). Experimental Gingivitis in Man. J. Periodontol..

[19] Swami V., Vijayaraghavan V., Swami V. (2016). Current trends to measure implant stability. J. Indian Prosthodont. Soc..

[20] Blazsek J., Dobo Nagy C., Blazsek I., Varga R., Vecsei B., Fejerdy P., Varga G. (2009). Aminobisphosphonate stimulates bone regeneration and enforces consolidation of titanium implant into a new rat caudal vertebrae model. Pathol. Oncol. Res..

[21] Brunski J.B., Puleo D.A., Nanci A. (2000). Biomaterials and biomechanics of oral and maxillofacial implants: Current status and future developments. Int. J. Oral Maxillofac. Implant..

[22] Carvalho C.M., Carvalho L.F., Costa L.J., Sa M.J., Figueiredo C.R., Azevedo A.S. (2010). Titanium implants: A removal torque study in osteopenic rabbits. Indian J. Dent. Res..

[23] Bernhardt R., Kuhlisch E., Schulz M.C., Eckelt U., Stadlinger B. (2012). Comparison of bone-implant contact and bone-implant volume between 2D-histological sections and 3D-SRmicroCT slices. Eur. Cell Mater..

[24] Bissinger O., Probst F.A., Wolff K.D., Jeschke A., Weitz J., Deppe H., Kolk A. (2017). Comparative 3D micro-CT and 2D histomorphometry analysis of dental implant osseointegration in the maxilla of minipigs. J. Clin. Periodontol..

[25] Davies J.E. (2007). Bone bonding at natural and biomaterial surfaces. Biomaterials.

[26] Rodrigo D., Aracil L., Martin C., Sanz M. (2010). Diagnosis of implant stability and its impact on implant survival: A prospective case series study. Clin. Oral Implants Res..

[27] Atsumi M., Park S.H., Wang H.L. (2007). Methods used to assess implant stability: Current status. Int. J. Oral Maxillofac. Implant..

[28] Huwiler M.A., Pjetursson B.E., Bosshardt D.D., Salvi G.E., Lang N.P. (2007). Resonance frequency analysis in relation to jawbone characteristics and during early healing of implant installation. Clin. Oral Implants Res..

[29] Sălăvăstru D.I., Gherghiţă O.R. (2020). Considerations regarding the use of experimental animal models in dental medicine—A literature review. AgroLife Sci. J..

[30] Hartung T. (2010). Comparative analysis of the revised directive 2010/63/E.U. for the protection of laboratory animals with its predecessor 86/609/E.E.C.—A t4 report. Altex.

[31] Ancuta D.L., Coman C., Alexandru D.M., Crivineanu M. (2020). Animal models used in testing the biocompatibility of the dental implant—A review. Bull. UASVM Vet. Med..

[32] (2016). Dentistry—Preclinical Evaluation of Dental Implant Systems—Animal Test Methods.

[33] Socransky S.S., Haffajee A.D., Cugini M.A., Smith C., Kent R.L. (1998). Microbial complexes in subgingival plaque. J. Clin. Periodontol..

[34] Freire M.O., Sedghizadeh P.P., Schaudinn C., Gorur A., Downey J.S., Choi J., Chen W., Kook J., Chen C., Goodman S.D. (2011). Development of an animal model for Aggregatibacter actinomycetemcomitans biofilm-mediated oral osteolytic infection: A preliminary study. J. Periodontol..

[35] Koutouzis T., Eastman C., Chukkapalli S., Larjava H., Kesavalu L. (2017). A Novel Rat Model of Polymicrobial Peri-Implantitis: A Preliminary Study. J. Periodontol..

[36] Clark D., Nakamura M., Miclau T., Marcucio R. (2017). Effects of aging on fracture healing. Curr. Osteoporos. Rep..

[37] Stegen S., Van Gastel N., Carmeliet G. (2015). Bringing new life to damaged bone: The importance of angiogenesis in bone repair and regeneration. Bone.

[38] Lang N.P., Berglundh T. (2011). Periimplant diseases: Where are we now?—Consensus of the Seventh European Workshop on Periodontology. J. Clin. Periodontol..

[39] Quirynen M., Bollen C.M. (1995). The influence of surface roughness and surface-free energy on supra- and subgingival plaque formation in man. A review of the literature. J. Clin. Periodontol..

[40] Sanz M., Alandez J., Lazaro P., Calvo J.L., Quirynen M., Van Steenberghe D. (1991). Histo-pathologic characteristics of peri-implant soft tissues in Branemark implants with 2 distinct clinical and radiological patterns. Clin. Oral Implants Res..

[41] Rovin S., Costich E.R., Gordon H.A. (1966). The influence of bacteria and irritation in the initiation of periodontal disease in germfree and conventional rats. J. Periodontal Res..

[42] Bezerra Bde B., Andriankaja O., Kang J., Pacios S., Bae H.J., Li Y., Graves D.T. (2012). *A. actinomycetemcomitans*-induced periodontal disease promotes systemic and local responses in rat periodontium. J. Clin. Periodontol..

[43] Hyde E.R., Luk B., Cron S., Kusic L., Mccue T., Bauch T., Bryan N.S. (2014). Characterization of the rat oral microbiome and the effects of dietary nitrate. Free Radic. Biol. Med..

[44] Ancuța D.L., Ioniță F., Alexandru D.M., Crivineanu M., Coman C. (2021). The rat and the sheep, animal models for the study of periodontitis and induced periimplantitis of bacterial strains specific to human oral microbiote. Lucr. Științifice Med. Vet. USAMV “Ion Ionescu De La Brad”.

[45] Shui Y., Li M., Su J., Chen M., Gu X., Guo W. (2021). Prognostic and clinicopathological significance of systemic immune-inflammation index in pancreatic cancer: A meta-analysis of 2365 patients. Aging.

[46] Hamad D.A., Aly M.M., Abdelhameid M.A., Ahmed S.A., Shaltout A.S., Abdel-Moniem A.E., Ragheb A.M.R., Attia M.N., Meshref T.S. (2021). Combined blood indexes of systemic inflammation as a mirror to admission to intensive care unit in COVID-19 patients: A multicentric study. J. Epidemiol. Glob. Health.

[47] Du Y.N., Chen Y.J., Zhang H.Y., Wang X., Zhang Z.F. (2021). Inverse association between systemic immune-inflammation index and bone mineral density in postmenopausal women. Gynecol. Endocrinol..

[48] Chaushu L., Tal H., Sculean A., Fernandez-Tome B., Chaushu G. (2020). Peri-implant disease affects systemic complete blood count values-an experimental in vivo study. Clin. Oral Investig..

[49] Chaushu L., Tal H., Sculean A., Fernandez-Tome B., Chaushu G. (2021). Effects of peri-implant infection on serum biochemical analysis. J. Periodontol..

